# Neuroregenerative Effects of Radio Electric Asymmetric Conveyer (REAC) Neuroregenerative (RGN-N) Therapy in Pediatric Adrenoleukodystrophy: A Case Report

**DOI:** 10.7759/cureus.74197

**Published:** 2024-11-22

**Authors:** Vania Fontani, Arianna Rinaldi, Salvatore Rinaldi

**Affiliations:** 1 Department of Regenerative Medicine, Rinaldi Fontani Institute, Florence, ITA; 2 Department of Adaptive Neuro Psycho Physio Pathology and Neuro Psycho Physical Optimization, Rinaldi Fontani Institute, Florence, ITA; 3 Research Department, Rinaldi Fontani Foundation, Florence, ITA

**Keywords:** adrenoleukodystrophy, cellular bioelectrical modulation, cognitive improvement, functional recovery, motor function recovery, neuroregenerative treatment, pediatric neurodegeneration, rare neurodegenerative disorders, reac neuroregenerative therapy, spasticity reduction

## Abstract

This case report presents the use of radio electric asymmetric conveyer (REAC) neuroregenerative (RGN-N) therapy in a pediatric patient with adrenoleukodystrophy (ALD), a progressive neurodegenerative disorder with limited therapeutic options. The patient underwent three REAC RGN-N treatment cycles, each lasting 72 hours, with approximately 6-7 hours of daily sessions. An asymmetric conveyor probe (ACP) was positioned along the spine to channel the interaction of the emitted radio electric field with cellular electro-metabolic alterations, promoting progressive bioelectrical restoration. The treatment parameters were pre-set on the REAC device (BENE 110, ASMED, Scandicci, Italy) and could not be altered by the operator, ensuring consistent therapeutic delivery. Significant functional improvements were observed across the motor, cognitive, and swallowing domains, as assessed by standardized scales. This report aligns with preclinical studies on REAC technology's potential for neuroregeneration and suggests REAC RGN-N therapy as a promising adjunctive intervention in ALD management.

## Introduction

Adrenoleukodystrophy (ALD) is an X-linked genetic disorder [1] characterized by the accumulation of very-long-chain fatty acids in the brain and adrenal cortex [2], leading to demyelination, neuroinflammation, and progressive neurological decline [3,4]. Available treatments, such as bone marrow transplantation [5] and pharmacological interventions, largely focus on delaying disease progression and managing symptoms without halting or reversing neurodegeneration [6]. Thus, there is a critical need for therapies that address both neurodegeneration and neuroinflammation in ALD, particularly for pediatric patients with rapid disease progression.

Recent studies in neuroregenerative medicine have explored the role of bioelectrical modulation in promoting neuroplasticity and functional recovery [7]. The radio electric asymmetric conveyer (REAC) technology, a non-invasive therapy delivering radio electric fields asymmetrically conveyed, has shown promise in inducing neuroregenerative responses [8-10]. Studies have demonstrated that REAC treatment upregulates neurogenic markers (e.g., neurogenin-1 and beta-3 tubulin) and reduces neuroinflammatory markers such as IL-1β and TNF-α, indicating its potential to stabilize neural circuits, mitigate inflammation, and support functional recovery​ [11,12]. This case report describes the application of REAC neuroregenerative (RGN-N) therapy in a pediatric ALD patient, with a focus on quantifying motor, cognitive, and swallowing improvements.

## Case presentation

Patient information

The patient, born on August 6, 2014, was diagnosed with ALD following an observed rapid decline in vision, motor skills, and cognitive function. After returning to school post-pandemic, teachers reported signs of hyperactivity and reluctance to participate in activities, prompting a referral to a psychologist, although no notable behavioral changes were identified during therapy. At home, subtle strabismus was noted, though consultations with two ophthalmologists confirmed no presence of strabismus. On April 16, 2021, the family sought emergency care due to the patient's worsening ability to see objects and frequent falls. Hospital evaluations, including computed tomography (CT) and magnetic resonance imaging (MRI), confirmed significant neurological damage consistent with ALD, with an initial Loes score [13] of 16. By mid-2021, his symptoms had advanced to severe dysphagia, spasticity, and cognitive impairment, necessitating comprehensive supportive care, including a gastrostomy for nutritional support.

Therapeutic interventions and course of treatment

**Figure 1:**
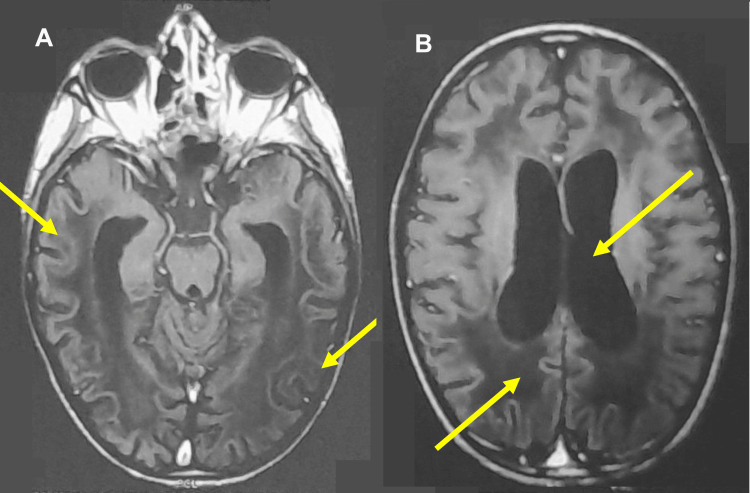
MRI scans illustrating the neurological impact of ALD on the brain structure of the pediatric patient. The scans emphasize progressive demyelination, (A) cortical atrophy, and (B) ventricular enlargement (indicated by yellow arrows), all hallmark features of ALD. MRI: magnetic resonance imaging; ALD: adrenoleukodystrophy

In June 2021, the patient received a bone marrow transplant, with his father as a partially compatible donor. The procedure was completed without immediate complications, and initially, the patient maintained stable motor and cognitive function, remaining able to walk, run, eat, and communicate. However, by late 2022, his condition deteriorated, marked by severe dysphagia, aphasia, cognitive decline, malnutrition, graft-versus-host disease, and a COVID-19 infection (Figure 1).

Due to impaired swallowing, a gastrostomy was placed to provide nutritional support. Concurrently, he experienced increased spasticity, sleep disturbances, persistent constipation, and frequent episodes of crying, indicative of physical discomfort and cognitive distress.

To address these symptoms, he was prescribed risperidone (1 ml nightly) to help stabilize mood and behavior, baclofen (10 mg every eight hours) for spasticity, prednisolone (1 ml every 12 hours) as an anti-inflammatory, and cannabidiol, starting at six drops every 12 hours and gradually increased to 12 drops. Despite this regimen, symptom relief remained limited.

In November 2023, REAC RGN-N therapy was initiated as a new approach aimed at supporting neurological recovery and enhancing functional outcomes beyond existing pharmacological and supportive care. Subsequent cycles of REAC RGN-N therapy were administered in April and September 2024, accompanied by motor physiotherapy, equine therapy, and speech therapy to support functional capacity.

Each of the three REAC RGN-N treatment cycles spanned 72 hours, with daily sessions lasting approximately 6-7 hours. During these sessions, an asymmetric conveyor probe (ACP) was positioned along the spine. The ACP does not deliver the therapeutic field directly; rather, it asymmetrically channels the interaction between the radio electric field emitted by the REAC device (BENE 110 ASMED, Scandicci, Italy) and the patient's cellular electro-metabolic alterations, facilitating progressive bioelectrical balance restoration. The device's parameters are pre-set and non-modifiable by the operator, ensuring consistency and standardization across all sessions.

Follow-up and outcomes

After the first cycle of REAC RGN-N therapy in November 2023, the patient demonstrated measurable improvements in multiple functional domains, which were carefully quantified using validated clinical scales to ensure objective assessment. These scales included the Functional Oral Intake Scale (FOIS) [14], Trunk Control Measurement Scale (TCMS) [15], Modified Ashworth Scale (MAS) [16], and Levels of Cognitive Functioning Scale (LCFS) [17], providing a standardized approach to tracking the patient's functional progress.

Swallowing and oral intake

At the outset of therapy, the patient relied exclusively on gastrostomy feeding, which corresponds to level 1 on the FOIS. Following the initial REAC cycle, he advanced to level 4, as he regained the ability to safely consume pureed foods orally, a significant development that reflects improved neuromuscular control over swallowing. This improvement in FOIS scores remained stable over subsequent cycles, suggesting sustained gains in oropharyngeal coordination and muscle function, essential for maintaining nutritional autonomy and reducing reliance on invasive feeding methods.

Motor control and trunk stability

The TCMS provided an essential evaluation of the patient's seated balance and postural control. Initially, he displayed poor trunk stability, limiting his ability to remain balanced in a seated position. Following the first REAC therapy cycle, improvements in dynamic sitting balance and trunk stability were observed. By the second cycle, these gains were maintained, and the patient demonstrated greater cervical control and a more stable posture, which were crucial for safe movement and interaction with his environment. These improvements in TCMS scores highlight REAC's possible effect on motor pathway stabilization, suggesting enhanced neuromuscular coordination and postural support.

Reduction in spasticity

Spasticity, a hallmark symptom of ALD's neurodegenerative progression, was quantitatively assessed using the MAS, where the patient initially scored 3-4, indicative of marked rigidity that impeded passive movement. After the first REAC cycle, spasticity levels decreased significantly, reaching a score of approximately 1+, which indicates only slight muscle resistance during movement. This reduction in MAS scores was sustained over the next cycles, allowing the patient to experience greater ease of movement, relaxation in the upper limbs, and reduced discomfort. These gains in motor flexibility suggest a favorable neuromodulatory effect of REAC therapy, potentially acting to normalize muscle tone and alleviate rigidity associated with neurodegenerative processes.

Cognitive engagement and communication

Cognitive improvements were tracked using the LCFS, revealing progressive advances in the patient's ability to interact and communicate purposefully. Initially, the patient showed limited response to external stimuli and verbal cues, likely corresponding to a lower LCFS level. After the first cycle, he exhibited increased attentiveness and was more responsive to verbal commands, suggesting cognitive gains that continued to develop with further cycles. By the third cycle, the patient achieved an LCFS level 4, indicative of purposeful responses and reliable engagement with caregivers through non-verbal communication methods, such as blinking. This cognitive progression underscores REAC therapy's potential impact on cortical and subcortical neural circuits, which may support improvements in attention, communication, and interactional abilities.

These functional gains, observed across swallowing, motor control, spasticity, and cognitive responsiveness, illustrate REAC RGN-N therapy's multi-dimensional impact on both motor and cognitive domains, aligning with preclinical findings suggesting neuroregenerative effects. Importantly, these improvements remained stable across treatment cycles, suggesting that REAC RGN-N therapy may provide a sustained benefit in neurodegenerative disorders like ALD, where functional deterioration is typically progressive. Such consistent outcomes highlight the value of REAC therapy in potentially altering the trajectory of neurodegenerative impairment, even in severe cases with limited therapeutic options.

## Discussion

The therapeutic outcomes in this case highlight REAC RGN-N therapy's potential role in addressing symptoms of neurodegeneration and neuroinflammation associated with ALD. The observed improvements in swallowing, trunk stability, and cognitive responsiveness suggest that REAC's bioelectrical modulation may support neuroplasticity and functional recovery. This aligns with preclinical findings that REAC RGN-N therapy upregulates neurogenic markers and reduces neuroinflammatory markers, indicating a potential mechanism through which REAC therapy may promote neural regeneration [11,18].

The unique mechanism of the ACP, which channels asymmetrically the REAC-emitted radio electric field to interact with cellular electro-metabolic alterations [7], facilitates bioelectrical stabilization and allows for targeted neuromodulation without operator-dependent variability. The fixed parameters of the BENE 110 device further ensure consistency in therapeutic application, which is essential in maintaining precision and therapeutic efficacy.

Given the rarity and diverse severity of ALD, this case report underscores the importance of in-depth case series and focused smaller-scale studies to further elucidate REAC RGN-N therapy's impact on neuroregeneration and functional recovery. Findings from detailed case reports are particularly valuable in rare disease contexts, providing insights that can guide therapeutic developments and individualized care approaches, even in the absence of large-scale trials. Future studies focused on neuroregenerative and neuroprotective pathways, as modulated by REAC technology, may provide essential knowledge for expanding therapeutic options in ALD and similar neurodegenerative disorders.

## Conclusions

This case report demonstrates the potential of REAC RGN-N therapy in facilitating functional recovery in a pediatric patient with ALD. The outcomes observed align with preclinical research suggesting REAC's role in promoting neuroregeneration and reducing inflammation, highlighting its potential as a supportive treatment option. Further studies are needed to validate these findings and assess REAC's applicability in neurodegenerative disease management.
